# Predictors of Stroke, Myocardial Infarction or Death within 30 Days of Carotid Artery Stenting: Results from the International Carotid Stenting Study

**DOI:** 10.1016/j.ejvs.2015.08.013

**Published:** 2016-03

**Authors:** D. Doig, E.L. Turner, J. Dobson, R.L. Featherstone, R.T.H. Lo, P.A. Gaines, S. Macdonald, L.H. Bonati, A. Clifton, M.M. Brown, ICSS Investigators

**Affiliations:** a Institute of Neurology, University College London, UK; b Department of Biostatistics and Bioinformatics, Duke University, Durham, NC, USA; c Duke Global Health Institute, Duke University, Durham, NC, USA; d Department of Medical Statistics, London School of Hygiene and Tropical Medicine, UK; e University Medical Centre, Utrecht, The Netherlands; f Sheffield Vascular Institute, Sheffield, UK; g Freeman Hospital, Newcastle Acute Hospitals NHS Foundation Trust, Newcastle, UK; h Department of Neurology and Stroke Unit, University Hospital Basel, Switzerland; i St George's Hospital, London, UK

**Keywords:** Carotid atherosclerosis, Carotid artery stenosis, Stroke, Myocardial infarction, Stents

## Abstract

**Objectives:**

Stroke, myocardial infarction (MI), and death are complications of carotid artery stenting (CAS). The effect of baseline patient demographic factors, processes of care, and technical factors during CAS on the risk of stroke, MI, or death within 30 days of CAS in the International Carotid Stenting Study (ICSS) were investigated.

**Methods:**

In ICSS, suitable patients with recently symptomatic carotid stenosis > 50% were randomly allocated to CAS or endarterectomy. Factors influencing the risk of stroke, MI, or death within 30 days of CAS were examined in a regression model for the 828 patients randomized to CAS in whom the procedure was initiated.

**Results:**

Of the patients, 7.4% suffered stroke, MI, or death within 30 days of CAS. Independent predictors of risk were age (risk ratio [RR] 1.17 per 5 years of age, 95% CI 1.01–1.37), a right-sided procedure (RR 0.54, 95% CI 0.32–0.91), aspirin and clopidogrel in combination prior to CAS (compared with any other antiplatelet regimen, RR 0.59, 95% CI 0.36–0.98), smoking status, and the severity of index event. In patients in whom a stent was deployed, use of an open-cell stent conferred higher risk than use of a closed-cell stent (RR 1.92, 95% CI 1.11–3.33). Cerebral protection device (CPD) use did not modify the risk.

**Conclusions:**

Selection of patients for CAS should take into account symptoms, age, and side of the procedure. The results favour the use of closed-cell stents. CPDs in ICSS did not protect against stroke.

**What this paper adds**

The International Carotid Stenting Study (ICSS) compared carotid artery stenting (CAS) with endarterectomy for stroke prevention in patients with recently symptomatic carotid artery stenosis. The aim of the present study was to determine if there were specific factors related to CAS procedures, the process of care, or baseline patient characteristics that significantly increased or decreased the risk of stroke, myocardial infarction, or death within 30 days of CAS in ICSS. It was found that increasing age independently increased the risk of CAS, while the risk was significantly lower in patients undergoing a right-sided procedure, in patients taking the combination of aspirin and clopidogrel, and in those presenting only with amaurosis fugax. Cerebral protection device use did not modify the risk, but the risk was significantly higher in patients treated with an open-cell stent compared to a closed-cell stent.

## Introduction

Carotid artery stenting (CAS) is an endovascular alternative to carotid endarterectomy (CEA) for the treatment of symptomatic atherosclerotic disease of the carotid artery. The International Carotid Stenting Study (ICSS) was a large randomized controlled open clinical trial that compared the efficacy and safety of CAS and CEA in patients with recently symptomatic carotid stenosis measuring greater than 50%. In an interim analysis, the risk of stroke, myocardial infarction (MI), or death within 30 days of the procedure was higher for those patients who received their allocated CAS procedure than for those who received their allocated CEA procedure (7.4% vs. 4.0%, *p* = .003).^1^ Haemodynamic disturbance, carotid embolism and thrombosis or occlusion of the carotid artery were all important mechanisms of ischaemic stroke in these patients.^2^

The optimal stenting technique, patient selection, and processes of care for CAS have yet to be determined. Endovascular carotid revascularization is a complex procedure requiring attention to pre-procedure medication, intra- and post-procedure haemodynamic control, stent design, and optimal medical therapy.^3^ The additional use of cerebral protection devices (CPDs), intended to catch debris arising from the plaque or dislodged thrombus to prevent distal embolization, has become more common in recent years^4^ despite conflicting evidence supporting their routine use.

The patients recruited to ICSS were examined to determine whether there are specific CAS procedures, processes of care or baseline patient characteristics associated with a higher risk of stroke, MI, or death within 30 days of the procedure.

## Methods

### Study design

#### Patient selection and protocol design

Details of the ICSS trial protocol are published elsewhere.^5^ In brief, clinically stable patients over 40 years old were eligible for randomization in ICSS if they had more than 50% recently symptomatic carotid stenosis suitable for either CAS or CEA. Patients were excluded if they had a major stroke with poor recovery, if their vascular anatomy rendered CAS or CEA unsuitable, if the stenosis was due to non-atheromatous disease, if cardiac bypass was planned within 1 month of the revascularization, or if there had been previous revascularization of the symptomatic artery.

Stenting was performed in accordance with strict protocol; the stents and protection devices used were required to be CE marked and approved by the trial steering committee, and a CPD was recommended whenever the operator thought one could safely be deployed. Centres and interventionists were accredited by the trial steering committee. Centres not fulfilling the trial protocol requirements for prior experience were permitted to join the trial as a “supervised” centre: procedures were supervised by a more experienced interventionist at these sites. The combination of the antiplatelet agents aspirin and clopidogrel prior to the procedure was recommended, and the administration of intra-procedural heparin was mandatory.

The Northwest Multicentre Research Ethics Committee in the UK approved ICSS. Participating centres obtained site-specific approval. Patients provided written informed consent.

#### Outcome events

A CAS technical data form was completed by investigators and returned to the central trial office. Stroke, MI, or death occurring within 30 days of the procedure was reported by investigators and confirmatory evidence was required to be submitted (brain imaging, ECG, cardiac enzymes, and/or death certificate where available). Stroke was defined as “an acute disturbance of focal neurological function with symptoms lasting more than 24 hours resulting from intracranial vascular disturbance”. A diagnosis of MI required two of the following criteria: cardiac enzymes more than twice the upper limit of normal, a history of chest discomfort for more than 30 minutes, or the development of specific ECG abnormalities. Outcome events were reported in detail to the central office by the local neurologist or stroke physician. Investigators at the trial office adjudicated the timing and cause of events by review of evidence either from witnesses or case note review at the centre involved. Major outcome events were submitted to an independent external adjudicator, who was masked to treatment allocation and who determined the cause, severity, and duration of the event. If this assessment differed from the initial assessment, a second external adjudicator reviewed the event and any differences were resolved by consensus.

#### Role of the funding source

The funders and sponsors of the study had no role in study design, data collection, data analysis, data interpretation, or the writing of this paper.

#### Statistical analysis

The data were analysed per-protocol, that is only CAS patients in ICSS in whom the randomly allocated procedure was initiated were included. A procedure was deemed to have been initiated if the patient underwent either local or general anaesthesia prior to commencement of the procedure. Patients who crossed over to CAS, received CAS after an attempt at endarterectomy or received medical therapy instead of CAS were excluded. Risk factors for stroke, MI, or death within 30 days of the procedure were examined using binomial regression. Patients with missing data were excluded from each relevant analysis. To examine the effects of the use of CPD type, stent design, and post-stent dilation only patients in whom a CPD or stent was deployed were analysed. A multivariable model was primarily developed using only those variables potentially available for all CAS patients (i.e. excluding cerebral protection device type, stent design and post-dilation variables) using a forward stepwise approach. A *p* value of <.05 was accepted as conferring statistical significance in all analyses. In sensitivity analysis, the multivariable model was reconstructed excluding patients in whom a stent was not deployed in order to be able to assess the effect of stent design and post-dilation variables, and by excluding patients in whom a CPD was not used to assess the effect of CPD type. Analyses were performed using Stata (StataCorp. 2011. Stata Statistical Software: Release 12. College Station, TX: StataCorp LP).

## Results

### Patient and procedure characteristics

**Figure 1:**
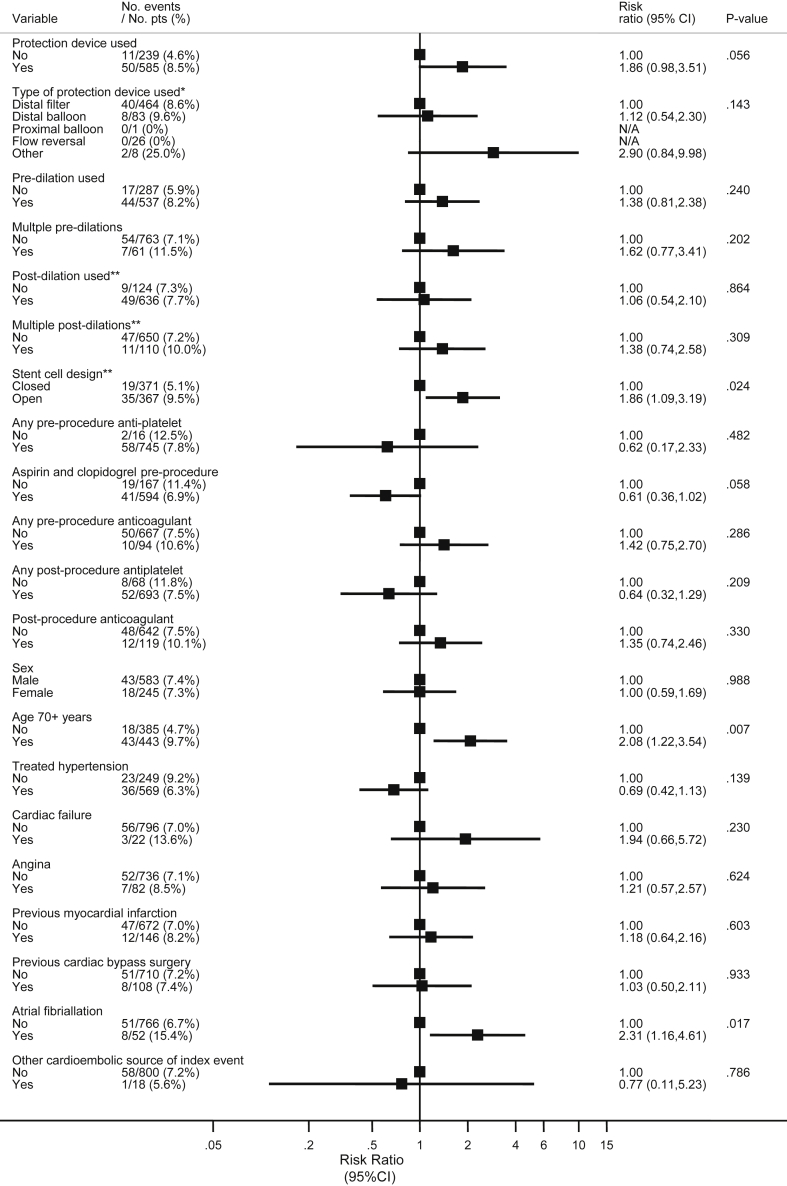

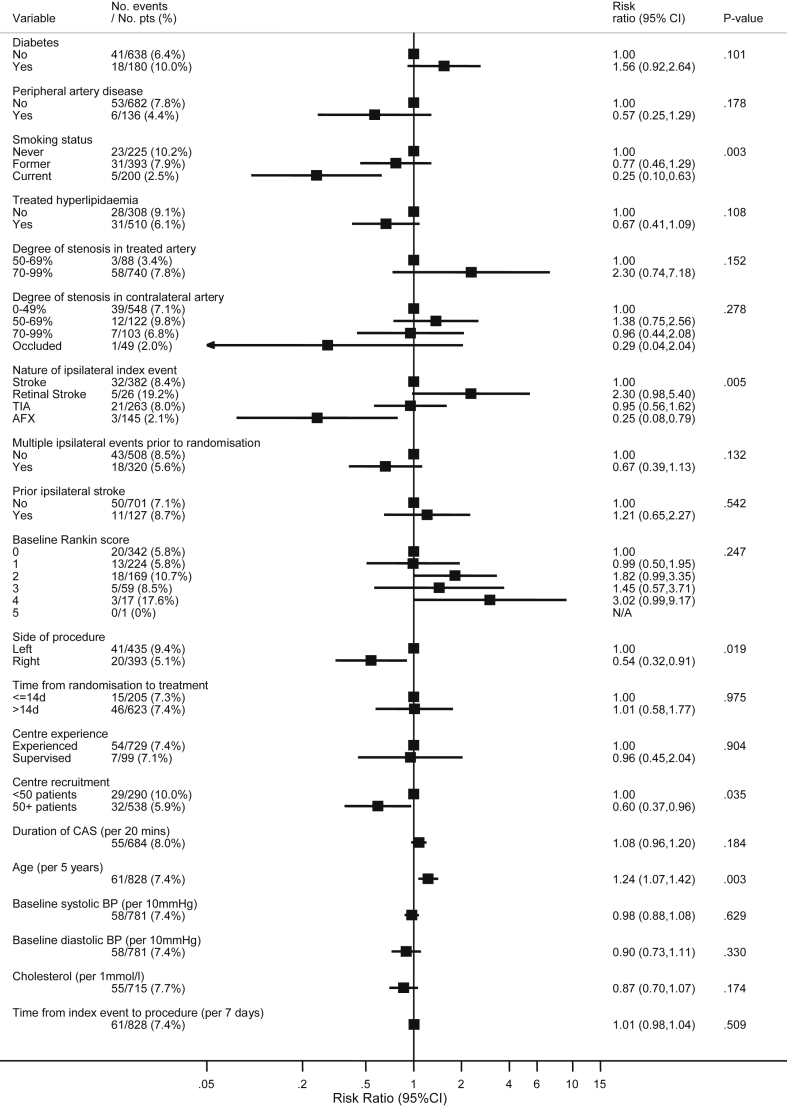
Univariable predictors of the risk of stroke, myocardial infarction or death within 30 days of carotid artery stenting in 828 ICSS per-protocol participants in whom the procedure was initiated.* Only patients with a protection device. ** Only patients were a stent was deployed. Patients with missing data were excluded for each relevant analysis. Variables with >1% missing data are: stent cell design (3%), any pre-procedure antiplatelet (8%), aspirin and clopidogrel pre-procedure (8%), any pre-procedure anticoagulant (8%), any post-procedure antiplatelet (8%), any post-procedure anticoagulant (8%), baseline Rankin score (2%), duration of CAS (17%), baseline systolic BP (6%), baseline diastolic BP (6%), and cholesterol (14%).

A total of 1,713 patients were randomized in ICSS. Of the 853 assigned to CAS, 828 procedures were initiated per protocol and were included in this analysis. There were more men (70.4%) than women and just over half of the patients were over the age of 70 (53.5%). Baseline characteristics of the patient are given in Fig. 1. Summary statistics for continuous variables in the analysis are detailed in Table 1. A stent was deployed in 764 of 816 procedures (92.2%) for which data were available. The type of stent was recorded in 752 of those procedures. In 367 (48.8%) procedures an open-cell stent was used, and in 371 (49.3%) a closed-cell stent was used. Individual stent types are listed in Table 2. A CPD was known to have been deployed in 585 (70.6%) procedures, of which 464 (79.3%) were known to be distal filter type devices; 71.7% of patients were known to be taking aspirin and clopidogrel in combination.

### Timing and cause of events up to 30 days after the procedure

Sixty-one of the 828 patients (7.4%) suffered stroke, MI, or death within 30 days of the procedure. The nature of the outcome events, the majority of which were stroke, are listed in more detail in Table 3. Forty-four of the 61 (72.1%) events occurred on the day of the procedure.

### Demographic and technical risk factors

The results of univariable analysis are presented in Fig. 1. The effect of age, analysed as a continuous variable in years, was an increase in risk of 1.24 for each 5 years (95% CI 1.07–1.42, *p* = .003). An increased risk of stroke, MI, or death was observed in patients with an open design of carotid stent (RR 1.86, 95% CI 1.09–3.19, *p* = .024) and in patients with atrial fibrillation (RR 2.31, 95% CI 1.16–4.61, *p* = .017). The risk was lower in patients who were current or former smokers at the time of enrolment compared with those who had never smoked (RR 0.25, 95% CI 0.10–0.63 and RR 0.77, 95% CI 0.46–1.29 respectively, global *p* = .003), and those with amaurosis fugax as the initial event prompting enrolment in the trial compared with those who suffered stroke as the index event (RR 0.25, 95% CI 0.08–0.79, global *p* = .005). The risk was also lower in those undergoing a right-sided procedure (RR 0.54, 95% CI 0.32–0.91, *p* = .019). Other demographic or technical factors did not significantly affect the risk. Patients in whom a CPD was used (*n =* 585) experienced a higher risk of events compared with those that did not (*n =* 239) (RR 1.86, 95% CI 0.98–3.1, *p* = .056), but this difference did not reach statistical significance. Patients treated in centres that enrolled more than 50 patients were at lower risk of an event (RR 0.60, 95% CI 0.37–0.96, *p* = .035), but the results in supervised centres (where procedures performed by more inexperienced interventionists were proctored) were not statistically different from those at experienced centres.

A multivariable model investigating variables potentially available for all CAS patients is presented in Table 4. Increasing age was found to increase the risk of stroke, MI, or death within 30 days of the procedure after adjustment for other variables in the model (RR 1.17 per 5 years of age, 95% CI 1.01–1.37, *p* = .039). The risk was decreased in right-sided procedures (RR 0.54, 95% CI 0.32–0.91, *p* = .020), in those patients taking aspirin and clopidogrel prior to the procedure (as compared with any other antiplatelet regimen, RR 0.59, 95% CI 0.36–0.98, *p* = .042), in smokers, and in those with a less severe index event. In the sensitivity analysis, the model was reconstructed excluding patients in whom a stent was not deployed. In this analysis, the use of an open-cell stent conferred higher risk (RR 1.92, 95% CI 1.11–3.33, *p* = .019). The results of this model are given in Table 5. The antiplatelet regimen was not adjusted for in this model as it was no longer a statistically significant predictor in this subset of patients after adjustment for the stent design variable (*p* = .30). There was no evidence of an association between CPD type and risk of an event in the sensitivity analysis excluding patients in whom a CPD was not used (data not shown). Patients whose procedure was carried out in an experienced centre, with a closed-cell stent and CPD, who received aspirin and clopidogrel anti-platelet therapy, and who were receiving treatment for hyperlipidaemia did not have a statistically significantly lower risk of the outcome than other patients (RR 0.61, 95% CI 0.28–1.32, *p* = .21).

## Discussion

### Summary

The risk of stroke, MI, or death within 30 days of CAS in ICSS was significantly higher in older patients, those undergoing a left-sided procedure, in non-smokers, in patients not taking the combination of aspirin and clopidogrel, and in those with a more severe index event. An analysis of patients in whom a stent was deployed demonstrated a doubling of the risk of the composite event in those receiving an open cell design stent compared with a closed cell design.

### Research in context

The study adds to emerging evidence that CAS is less safe in older patients. Individual patient data meta-analysis of the three large European trials of CAS versus CEA (ICSS, the Endarterectomy Versus Angioplasty in patients with Symptomatic Severe carotid Stenosis (EVA-3S) trial^6^ and the Stent-Protected Angioplasty versus Carotid Endarterectomy (SPACE) trial^7^ found that although risks of stroke or death within 120 days were similar in patients allocated to CAS or CEA in patients under the age of 70 years, the risk of CAS was twice that of CEA in patients over 70 with no reduction in the severity of events experienced by this age group.^8^ CREST showed a similar effect of age.^9^ It has now been shown that this effect is related to an increasing risk of stenting with increasing age. Age should therefore be taken into account when selecting patients for the procedure.

Stent design also had a strong influence on risk of stent deployment in other trials. A higher risk of ipsilateral stroke or ipsilateral stroke death was found in patients treated with an open cell stent design in the SPACE study (OR 2.13, 95% CI 1.07–3.76),^10^ and similar trends are found in large observational studies.^11^ Our current observations in the largest randomized trial of stenting for symptomatic stenosis confirm this finding. This effect of stent design may be more pronounced in symptomatic patients due to the unstable nature of recently complicated atheromatous plaque; closed-cell stents have a smaller open area between cell struts and therefore greater coverage of the atheromatous lesion.

Cerebral protection devices were introduced in the assumption that they would reduce cerebral embolism during stent deployment. A systematic review of the results of case series comparing older data with results after the introduction of CPDs appeared to confirm this assumption,^12^ but the results could also have reflected other improvements in technique and patient selection over time. In keeping with the latter possibility, the current analysis has shown no evidence that CPD use in ICSS protected against stroke, MI, or death within 30 days of the procedure. The results are in keeping with the results of the ICSS MRI substudy which demonstrated a 2.7-fold higher risk of new ischaemic brain lesions on post-treatment diffusion-weighted (DWI) brain MRI in patients treated in centres with a policy of CPD use.^13^ In contrast to these findings in ICSS, the EVA-3S trial issued a clinical alert after finding that the 30-day risk of stroke in patients undergoing unprotected CAS was nearly four times that of patients undergoing protected CAS in the first 80 patients to be randomized.^14^ However, this difference is unlikely to explained by the use of a CPD since only two strokes in those treated without a CPD occurred on the day of the procedure. Two small randomized trials of CAS with and without filter-type CPDs, which were the predominant type used in ICSS, have reported results. One trial in the United States showed no reduction in DWI-MRI lesions post-procedure in the CPD group,^15^ the other UK-based trial demonstrated an increase in both new ischaemic brain lesions post-procedure and procedural particulate microemboli as detected by trans-cranial Doppler in the CPD group.^16^ Newer types of devices, including flow reversal systems, might be more effective. However, the data do not support the routine use of a distal filter CPD during stenting procedures.

The reduction in 30-day neurological complications in patients taking ‘dual’ antiplatelet therapy has been described before,^17^ and reinforces the recommendation that all CAS patients receive aspirin and clopidogrel prior to the procedure where appropriate. The finding that current smoking was associated with a lower complication rate is difficult to explain and has not been described elsewhere. It is therefore possible that this is a chance finding or that there may be residual confounding.

The chosen combined outcome measure encapsulates serious complications that patients and doctors wish to avoid and was chosen to match the primary outcome measure used in the previous analysis of short-term outcomes in ICSS.^1^ However, some variables such as choice of stent design, might be expected to have a much greater impact on stroke rates than on MI or non-stroke death. In practice, the results of the analysis indicate the risk factors for stroke during CAS, given that were only three MI or non-stroke deaths compared to 58 stroke events included in the combined outcome event used.

### Limitations of the analysis

CAS is a rapidly developing procedure, and newer CPD and stent types have become available since the first patient enrolment in ICSS. In some patients, information regarding baseline risk factors was unavailable and some procedures were abandoned before the deployment of either a CPD or stent, limiting the inclusion of these patients and variables in a multivariable model. Multiple comparisons without statistical correction raise the possibility of obtaining a Type I (false positive) error. This is not a randomized comparison of stenting technique or perioperative processes of care, and it is possible that unmeasured confounders are associated with the risk of stroke, MI, or death. The limited number of events in the patient group limited the number of variables supported in a multivariable model.

## Conclusions

Selection of patients for CAS should take into account risk factors for peri-procedural stroke including symptoms, the patient's age and side of the procedure. Stenting should be used with caution in older patients. These results favour the use of closed-cell stent design, without the addition of a cerebral protection device, and reinforce the need for dual antiplatelet therapy with aspirin and clopidogrel. Increased power to further characterize groups of patients at higher risk or technical procedures associated with an increased complication rate will be facilitated through combined individual patient data analyses from recent randomized trials of carotid stenosis as part of the Carotid Stenosis Trialists Collaboration (http://www.carotid-trialists.com/).

## Figures and Tables

**Table 1:** Summary statistics for baseline age, systolic blood pressure, diastolic blood pressure, total cholesterol and time from event to index procedure in 828 patients undergoing CAS (mean [SD] unless otherwise stated).

Patient characteristic	No. events/no. patients^a^	Mean (SD) or median (IQR)
Baseline age in years	61/828	70 (9)
Baseline systolic blood pressure in mmHg	58/781	147 (24)
Baseline diastolic blood pressure in mmHg	58/781	79 (12)
Baseline total cholesterol in mmol/L	55/715	4.8 (1.3)
Time index event to procedure in days (median [IQR])	61/828	35 (15, 82)
Duration of CAS in minutes (median [IQR])	55/684	60 (49.5, 80)

IQR = interquartile range; SD = standard deviation.

a Patients with available data.

**Table 2:** Design and types of stent used in ICSS.

Design and type of stent	Number of patients
Stent deployed	764
Closed cell	371
Wallstent	318
XACT	48
Invatec	5
Open cell	367
Cordis	209
EV3	82
Acculink	70
XPONENT	3
NEXSTENT	3
Unknown type	26
Stent not deployed	64

**Table 3:** Type of outcome event occurring within 30 days of carotid stenting in patients who received their allocated procedure.

Any stroke	58^a^
Ischaemic stroke	56
Haemorrhagic stroke	2
Fatal myocardial infarction within 30 days	3^a^
Non-fatal myocardial infarction	0
Death unrelated to stroke or myocardial infarction	1

a One patient had both stroke and subsequent fatal myocardial infarction.

**Table 4:** Independent predictors of risk of stroke, MI or death within 30 days of carotid artery stenting in 748 ICSS per-protocol participants in whom the procedure was initiated and for whom complete predictor data are available. Results obtained from multivariable binomial regression.

Variable	No. events/no. patients^a^	Adjusted risk ratio (95% CI)	Global *p*
Age (per 5 years increase)	59/748	1.17 (1.01–1.37)	.039
**Smoking status**			
Never	23/207	1.00	.030
Former	31/359	0.86 (0.52–1.43)	
Current	5/182	0.33 (0.13–0.85)	
**Nature of ipsilateral index event**			
Stroke	31/347	1.00	.019
Retinal stroke	5/24	2.47 (1.11–5.52)	
TIA	20/242	0.99 (0.58–1.68)	
Amaurosis fugax	3/135	0.32 (0.10–1.02)	
**Side of procedure**			
Left	40/394	1.00	.020
Right	19/354	0.54 (0.32–0.91)	
**Antiplatelet regimen pre-procedure**			
Any other antiplatelet	19/165	1.00	.042
Aspirin and clopidogrel in combination	40/583	0.59 (0.36–0.98)	

a Patients with data available included in the multivariable model.

**Table 5:** Results of sensitivity analysis in 725 ICSS per-protocol participants in whom the procedure was initiated and a stent was deployed and for whom complete predictor data are available. Results obtained from multivariable binomial regression.

Variable	No. events/no. patients^a^	Adjusted risk ratio (95% CI)	Global *p*
Age (per 5 years increase)	52/725	1.17 (1.00–1.38)	.055
**Smoking status**			
Never	21/196	1.00	.024
Former	27/322	0.80 (0.47–0.38)	
Current	4/180	0.28 (0.10–0.81)	
**Nature of ipsilateral index event**			
Stroke	28/330	1.00	.013
Retinal stroke	4/22	2.33 (0.91–5.94)	
TIA	18/242	0.97 (0.55–1.70)	
Amaurosis fugax	2/131	0.22 (0.05–0.91)	
**Side of procedure**			
Left	34/378	1.00	.056
Right	18/347	0.59 (0.34–1.01)	
**Stent cell design**			
Closed	18/365	1.00	.019
Open	34/360	1.92 (1.11–3.33)	

a Patients with data available included in the multivariable model.
